# Circadian Deregulation in Multiple Myeloma: *BMAL1*/*CLOCK* Expression Patterns and Diagnostic Performance

**DOI:** 10.1111/ijlh.70154

**Published:** 2026-05-21

**Authors:** Hamide Albayrak, Mustafa Ertan Ay, Anıl Tombak, Kenan Çevik, Özlem İzci Ay, Tuba Kabasakal, Mehmet Emin Erdal

**Affiliations:** ^1^ Health Science Institute, Department of Medical Biology Mersin University Mersin Türkiye; ^2^ Faculty of Medicine, Department of Medical Biology and Genetics Mersin University Mersin Türkiye; ^3^ Faculty of Medicine, Department of Hematology Mersin University Mersin Türkiye

**Keywords:** *BMAL1*, circadian clock, *CLOCK*, diagnostic biomarkers, gene expression, multiple myeloma

## Abstract

**Background:**

Circadian clock disruption has emerged as a relevant axis in cancer; however, the expression patterns and diagnostic relevance of *BMAL1* and *CLOCK* in multiple myeloma (MM) remain insufficiently defined.

**Methods:**

*BMAL1* and *CLOCK* mRNA expression was quantified by RT‐qPCR in bone marrow samples from 46 newly diagnosed MM patients and 13 healthy controls. Group comparisons, ROC analyses, multivariate logistic regression, partial correlation, and machine‐learning models (logistic regression, random forest, and support‐vector machine) with stratified 5‐fold cross‐validation (including class‐weighted sensitivity analysis) were performed.

**Results:**

*BMAL1* expression was significantly higher in MM than in controls (1.12 ± 0.33 vs. 0.85 ± 0.33, *p* = 0.016; Cohen's *d* = 0.83), whereas *CLOCK* showed no significant univariate difference (*p* > 0.2). *BMAL1* demonstrated moderate diagnostic performance (AUC = 0.729), while *CLOCK* alone showed limited discrimination (AUC = 0.617). Combined analysis modestly improved performance (AUC = 0.756). In multivariate logistic regression, *BMAL1* remained a strong independent predictor of MM (OR = 3343.4, *p* = 0.0046), whereas *CLOCK* showed an inverse adjusted association (OR = 0.0037, *p* = 0.019). *BMAL1* and *CLOCK* expression were strongly correlated in MM (*ρ* ≈ 0.80), persisting after adjustment for age, ISS stage, and β2‐microglobulin (partial *r* = 0.847, *p* = 2.2 × 10–13). Among classifiers, the random forest achieved the best performance (AUC = 0.832; AUPRC = 0.952). Specificity was limited across classifiers, indicating that these performance estimates are exploratory and require validation in larger cohorts.

**Conclusion:**

MM is characterized by *BMAL1* upregulation within a preserved but imbalanced *BMAL1/CLOCK* axis rather than uniform shifts across circadian genes. Although diagnostic performance is moderate, consistent signals across statistical and machine‐learning approaches support an MM‐associated circadian expression pattern warranting further validation.

## 1 Introduction

MM is the second most frequent hematologic malignancy with an estimated 34 920 new cases in the United States in 2021 [1]. It is a complex, incurable illness characterized by uncontrolled proliferation of clonal plasma cells which results in an overabundance of immunoglobulins or immunoglobulin chains that are inactive [2, 3]. These immunoglobulins can accumulate in the body and interact with other cells in the bone marrow to cause a number of problems, including anemia, bone lesions, infections, hypercalcemia, renal failure, fatigue, and pain [3, 4]. Smoldering myeloma (SMM) and monoclonal gammopathy of undetermined significance (MGUS) are thought to be MM's pre‐malignant stages [5]. The age‐standardized incidence is approximately 5 cases per 100 000. The median age of patients at diagnosis is approximately 66–70 years, and 37% of patients are under 65 years of age. It is slightly more common in males [6]. Chromosomal translocations, aneuploidy, genetic mutations, and epigenetic abnormalities all play crucial roles in the occurrence and progression of the disease, but the pathophysiology of multiple myeloma (MM) is complex and highly heterogeneous [6, 7]. Recent genomic analyses further highlight that MM follows a dynamic evolutionary trajectory, with temporal shifts in clonal architecture shaping clinical course and treatment response [8]. Beyond chronological age, an immune age metric has recently been shown to predict outcomes in older MM patients receiving daratumumab‐based therapy, underscoring the importance of time‐related biological processes in disease behavior and treatment response [9]. Although significant progress in MM treatment has been achieved, there is an urgent need for developing new therapeutic approaches to improve the clinical outcome of MM patients.

The circadian clock is a homeostatic timekeeping system that regulates numerous physiological, biochemical, and metabolic processes, enabling organisms to adapt to the daily light–dark cycle through an approximately 24‐h rhythm that has evolved over millions of years [10, 11, 12]. In mammals, the circadian clock is controlled by a central pacemaker located in the suprachiasmatic nucleus of the hypothalamus (SCN) [11, 12]. Recent studies suggest that peripheral clocks can maintain circadian activity with limited guidance from the central pacemaker, suggesting the existence of an autonomous mechanism based on the interactions between cellular components [13]. At least 9 core and closely related clock components have been identified to regulate circadian rhythms in peripheral tissues, consisting of three period proteins (PER1, PER2, and PER3), two cryptochromes (CRY1 and CRY2), CLOCK (circadian locomotor output cycles kaput), NPAS2 (neuronal PAS domain protein 2), Casein kinase1 ε (CK1ε), and BMAL1 (brain and muscle ARNT‐like protein 1) [11, 12]. As central players in the circadian clock molecular machinery, BMAL1 and CLOCK can form heterodimer complexes with each other and drive transcription from E‐box elements located in the promoter regions of circadian‐responsive genes. Period and cryptochrome proteins negatively regulate CLOCK/BMAL1 dimer‐mediated transcription and thereby generate the feedback loop that controls the timing of clock gene transcription [11, 12].

Recent experimental studies have demonstrated that the circadian clock machinery directly intersects with oncogenic signaling, cell‐cycle progression, DNA repair, apoptosis, metabolism, and immune regulation [11, 12]. Disruption of this system promotes several hallmark cancer phenotypes, including uncontrolled proliferation, impaired apoptosis, metastatic potential, immune evasion, angiogenesis, and resistance to anticancer therapy. Experimental models provide compelling evidence for this link: loss or suppression of BMAL1 or CLOCK accelerates tumor growth in colon cancer, breast cancer, hepatocellular carcinoma, pancreatic cancer, and non–small cell lung carcinoma, whereas restoring circadian rhythm components can reduce tumor burden [10, 11, 12, 13, 14, 15, 16]. In hematologic malignancies, clock gene dysregulation has similarly been implicated in altered cell‐cycle dynamics and survival pathways, as reported in chronic lymphocytic leukemia and other B‐cell neoplasms [12, 17]. A recent commentary further emphasized that circadian deregulation represents a biologically meaningful axis in B‐cell tumors, shaping both tumor evolution and treatment vulnerability [18]. Collectively, these findings highlight the broader oncogenic impact of circadian disruption and motivate its exploration in multiple myeloma.

Among the negative‐limb components of the circadian oscillator, PER3 variants have been implicated in sleep regulation and have also been investigated in cancer susceptibility [11, 12]. Recent findings have also begun to implicate circadian dysregulation directly in MM biology. A recent genetic study reported that polymorphisms in the PER3 clock gene may influence susceptibility to MM, suggesting that alterations in circadian regulation could contribute to disease onset [19]. Additionally, pharmacologic activation of the circadian regulator REV‐ERB with the synthetic agonist SR9009 has been shown to exert synergistic antitumor effects in MM cells by suppressing GRP78‐dependent autophagy and lipogenesis [20]. Together, these studies provide early evidence that circadian pathways may play a functional role in MM development and therapeutic vulnerability.

However, despite these emerging data, the expression patterns of the core positive‐limb genes BMAL1 and CLOCK in MM bone marrow, and their potential diagnostic relevance, have not been systematically evaluated. Thus, we investigated the mRNA expression of *BMAL1* and *CLOCK* in newly diagnosed MM patients and healthy controls, examined their association with clinical parameters, and explored their diagnostic potential using classical statistical methods and machine‐learning approaches.

## 2 Materials and Methods

### 2.1 Patients and Samples

The present case–control study has been approved by Institutional Health Sciences Ethics Committee (approval number 2020/123) and was in compliance with the guidelines of the Declaration of Helsinki. All participants signed written informed consent. A total of 46 newly diagnosed, previously untreated MM patients were enrolled. An a priori power analysis indicated that the available sample size was sufficient to detect large between‐group effects; however, smaller effects—particularly for *CLOCK*—may remain underpowered given the limited number of controls. The following clinicopathological parameters were collected for all patients: age, gender, ISS‐R (Revised international staging system) stages, Durie‐Salmon stages, LDH (lactate dehydrogenase) levels, Ig (immunoglobulin) types, β2‐MG (Beta2 micro‐globulin), CRP (C‐reactive protein), albumin, creatinine, hemoglobin, calcium levels, and heavy/light chain types were recorded. The clinicopathological characteristics of the 46 patients with MM studied in the present study are summarized in Table 1.

**TABLE 1:** Basic characteristics of patient and control group.

Variable	Category	MM patients (*n* = 46)	Controls (*n* = 13)
Sex	Male	27 (58.7%)	8 (61.5%)
	Female	19 (41.3%)	5 (38.5%)
Age	≤ 65 years	25 (54.3%)	7 (53.8%)
	> 65 years	21 (45.7%)	6 (46.2%)
ISS‐R stage	Stage I	6 (13.0%)	ND
	Stage II	34 (73.9%)	ND
	Stage III	6 (13.0%)	ND
Durie‐Salmon	Stage I	16 (34.8%)	ND
	Stage II	20 (43.5%)	ND
	Stage III	10 (21.7%)	ND
β2‐MG (mg/L)	< 3.5	18 (39.1%)	ND
	3.5–5.5	19 (41.3%)	ND
	> 5.5	9 (19.6%)	ND
Albumin	Normal	23 (50.0%)	ND
	Low	23 (50.0%)	ND
LDH	Normal	20 (43.5%)	ND
	High	17 (37.0%)	ND
	Low	9 (19.6%)	ND
CRP (mg/L)	≤ 5	22 (47.8%)	ND
	> 5	24 (52.2%)	ND
Calcium	Normal	34 (73.9%)	ND
	High	3 (6.5%)	ND
	Low	9 (19.6%)	ND
Creatinine	≤ 0.9 mg/dL	20 (43.5%)	ND
	> 0.9 mg/dL	26 (56.5%)	ND
Hemoglobin	Normal	24 (52.2%)	ND
	Low	22 (47.8%)	ND
Ig heavy chain	IgG	37 (80.4%)	ND
	IgA	9 (19.6%)	ND
Ig light chain	κ	32 (69.6%)	ND
	λ	14 (30.4%)	ND

*Note:* Values are shown as number (%). Clinical staging and laboratory parameters were assessed only in MM patients. ND, not defined.

Abbreviations: CRP, C‐reactive protein; Ig, immunoglobulin; ISS‐R, Revised International Staging System; LDH, lactate dehydrogenase; β2‐MG, beta‐2 microglobulin.

The control group consisted of 13 individuals without known hematological or malignant disorders. To reduce baseline confounding, controls were screened to exclude active infection, known malignancy/hematologic disease, and chronic inflammatory/autoimmune disorders; nevertheless, cardiovascular disease and perioperative physiological stress are inherent to this population and were considered when interpreting baseline expression levels. Because bone marrow aspiration cannot ethically be performed in healthy volunteers, control samples were obtained intraoperatively during open‐heart surgery, where a minimal amount of sternal marrow is incidentally aspirated for clinical access and would otherwise be discarded. Only age and sex were available for the control group due to the intraoperative nature of sampling.

Bone marrow samples from all participants were collected between 9 and 10 a.m. to limit the effects of time‐dependent expression changes. All participants were under hospital medical supervision before sampling. Nevertheless, we did not prospectively record individual sleep–wake schedules, fasting duration, or peri‐procedural sedative/anesthetic exposure. Accordingly, our standardized 9–10 a.m. sampling should be interpreted as a controlled snapshot designed to reduce between‐subject timing variability, rather than as a direct assessment of circadian rhythmicity across the day. In this manuscript, “circadian deregulation” refers to cross‐sectional between‐group differences in normalized mRNA levels measured at a fixed morning time point, and does not demonstrate a phase shift or loss of rhythmic oscillation. Accordingly, our design evaluates between‐group differences at a standardized time point, not diurnal phase or amplitude parameters.

### 2.2 RNA Isolation, cDNA Synthesis, and Gene Expression Analysis by Real‐Time Quantitative PCR

Total RNA of each sample was extracted from bone marrow cells using TRIzol reagent (Invitrogen, Carlsbad, CA, USA). Total RNA was extracted from bulk bone marrow aspirates; therefore, measured transcript levels reflect aggregate expression across malignant plasma cells and non‐malignant marrow components. Cell‐type–resolved validation (e.g., CD138+ enrichment) was not feasible within the current project scope and is planned for future work. RNA purity was confirmed by OD260/280 ratio (1.8–2.0). RT‐PCR conditions for both genes in a final reaction volume of 50 μL consisted of a total of 2 μg RNA (used per reaction), 200 U/μL Revertaid Reverse Transcriptase (Thermo Scientific, Vilnius, Lithuania), 5 × RT buffer, poly‐T primer, 2 mM each of dNTPs, 40 U/μL of RiboLock RNase inhibitor (Thermo Scientific, Vilnius, Lithuania) and nuclease‐free water. Thermal cycling conditions included 60 min at 37°C, 5 min at 95°C and then held at 4°C. Obtained cDNAs were stored at −20°C until Real‐time PCR amplification. The forward and reverse primers and TaqMan probe sequences specific to *BMAL‐1*, *CLOCK*, and *β‐actin (ACTB)* were designed using the Primer Express 3.0 software (Applied Biosystems, Foster City, CA, USA) and are listed in Table S1. To ensure target specificity and isoform compatibility while avoiding SNP regions, candidate oligonucleotides were evaluated using NCBI BLAST (version 2.14.0, National Center for Biotechnology Information, Bethesda, MD, USA, accessed May 2023). Only highly specific sequences were synthesized. Assay validation was performed using Total RNA Control (Cat. No. 4307281, Applied Biosystems, Foster City, CA, USA), and any non‐reproducible primer–probe sets were excluded. Primer efficiency was between 90% and 110%. *β‐actin* was used as an endogenous control to validate selected genes. While *β‐actin* was selected as the reference gene based on previous hematologic studies, possible variability within bone marrow tissue is acknowledged as a methodological limitation of the study.

Real‐time quantitative PCR was performed in the ABI Prism 7500 Real‐Time PCR System (Applied Biosystems, Foster City, CA, USA; Thermo Fisher Scientific Inc.) and the PCR cycling parameters were set as follows: after pre‐incubation at 50°C for 2 min, denaturation at 95°C for 10 min, followed by 50 cycles of 95°C for 15 s, 60°C for 1 min. Relative gene expression was calculated using the 2^−ΔΔCt^ method. All qPCR reactions were performed in duplicate, and specificity was ensured by the absence of nonspecific amplification in negative controls and by uniform amplification kinetics across samples.

### 2.3 Statistical Analysis

Gene expression levels of *BMAL1* and *CLOCK* were quantified using the 2^−ΔΔCt^ method. Group comparisons between Multiple Myeloma (MM) patients and healthy controls were performed using Welch's *t*‐test or the Mann–Whitney *U* test depending on normality (Shapiro–Wilk). Effect sizes (Cohen's *d*) and distributional metrics (median, IQR) were calculated to characterize the magnitude of group differences.

To examine the diagnostic value of each transcript, ROC analysis was conducted and area under the curve (AUC) values were computed. A multivariate logistic regression model was used to evaluate the combined and independent contributions of *BMAL1* and *CLOCK* expression to MM risk. Because *BMAL1* and *CLOCK* were strongly correlated, multicollinearity was anticipated; therefore, the multivariable coefficients were interpreted as exploratory and not as independent biological effects. In particular, the sign of the adjusted *CLOCK* coefficient should be interpreted as the residual (*BMAL1*‐independent) component of *CLOCK* within the joint model rather than as evidence of global *CLOCK* downregulation. Model performance was visualized using forest plots for odds ratios and calibration curves to assess goodness of fit.

The association between gene expression and clinical/laboratory characteristics (age, sex, ISS stage, Durie–Salmon stage, β_₂_‐microglobulin, CRP, calcium, LDH, creatinine, hemoglobin, immunoglobulin type, and light‐chain class) was evaluated using the Mann–Whitney *U* test or Kruskal–Wallis tests.

To test whether the relationship between *BMAL1* and *CLOCK* expression was independent of major prognostic variables, a partial correlation analysis was performed while controlling for age, ISS stage, and β_₂_‐microglobulin categories. Residual‐based scatter plots with 95% confidence ellipses were used to visualize adjusted inter‐gene coupling.

Finally, to assess the diagnostic utility of the circadian two‐gene signature, three supervised machine‐learning models—logistic regression, random forest, and support‐vector machine (RBF kernel)—were trained using stratified 5‐fold cross‐validation. Model performance was summarized using ROC curves (AUC), precision‐recall (PR) curves (AUPRC, average precision), accuracy, sensitivity, specificity, precision, and Brier scores. All analyses were performed using standard statistical and machine‐learning libraries in Python. Given the class imbalance (46 MM vs. 13 controls), machine‐learning analyses were treated as hypothesis‐generating and were interpreted together with specificity, calibration, and precision–recall metrics rather than as a ready‐to‐use diagnostic tool. We did not apply synthetic oversampling/undersampling because of the small sample size and the risk of information leakage; instead, we emphasized precision–recall curves, specificity, and calibration, and we frame these models as hypothesis‐generating. As a sensitivity analysis for class imbalance, we repeated stratified 5‐fold cross‐validation using class‐weighted versions of each classifier (logistic regression and SVM: class_weight = “balanced; random forest: class_weight” = “balanced_subsample”).

### 2.4 Use of Artificial Intelligence Tools

An artificial intelligence‐based language model (ChatGPT, OpenAI, USA) was used solely to assist with English language refinement, grammatical corrections, and stylistic clarity of the manuscript text. The AI tool was not used to generate scientific content, interpret data, perform statistical analyses, or influence study design or conclusions. All analyses, results, and interpretations were conceived, conducted, and verified exclusively by the authors. The AI tool did not meet the criteria for authorship and is therefore not listed as an author.

## 3 Results

### 3.1 *BMAL1* and *CLOCK* Gene Expression in Multiple Myeloma and Controls

**FIGURE 1:**
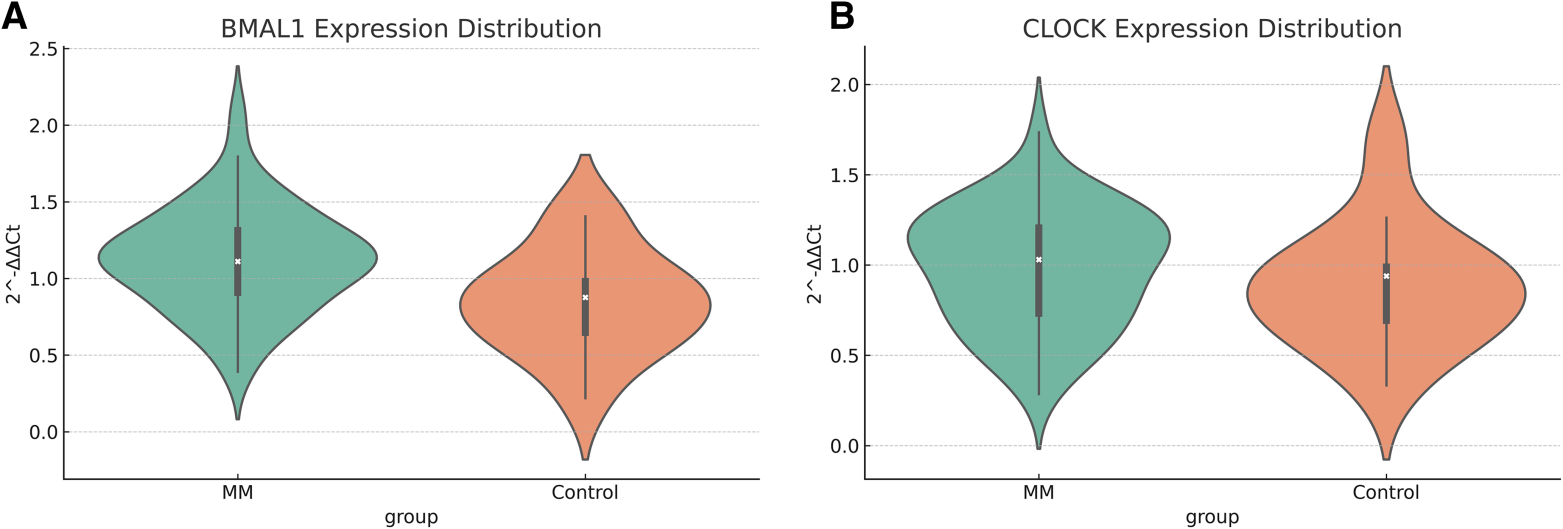
Relative expression levels of *BMAL1* (A) and *CLOCK* (B) genes in multiple myeloma (MM) patients and healthy controls. Violin plots display the distribution of normalized expression values (2^−ΔΔCt^) for each group, with median and interquartile ranges indicated. Statistical comparisons were performed using the Mann–Whitney *U* test (*p* < 0.05 considered significant).

Quantitative RT‐PCR analysis revealed distinct expression profiles of the core circadian genes *BMAL1* and *CLOCK* in Multiple Myeloma (MM) patients compared with healthy controls (Figure 1, Table 2). *BMAL1* expression levels (2^−ΔΔCt^) were significantly higher in MM patients (mean ± SD = 1.12 ± 0.33) than in controls (0.85 ± 0.33; Welch's *t* = 2.64, *p* = 0.016; Mann–Whitney U, *p* = 0.012), with a large effect size (Cohen's *d* = 0.83). In contrast, *CLOCK* expression showed only a modest increase in MM (0.98 ± 0.33) compared with controls (0.88 ± 0.34), which did not reach statistical significance (*t*‐test *p* = 0.351; Mann–Whitney *p* = 0.204). Shapiro–Wilk tests did not indicate significant deviation from normality for either transcript (*p* > 0.29).

**TABLE 2:** Comparison of *BMAL1* and *CLOCK* gene expression between multiple myeloma and control groups.

Gene group *n*	Mean (2^−ΔΔCt^)	SD	Median [IQR]	Shapiro–Wilk p	*t*‐test *p*	Mann–Whitney *p*	Cohen's *d*	AUC (ROC)
*BMAL1* Control *n* = 13	0.845	0.334	0.78 [0.63–1.05]	0.734	0.016	0.012	0.83	0.729
*BMAL1* MM *n* = 46	1.121	0.333	1.09 [0.91–1.31]	0.825				
*CLOCK* Control *n* = 13	0.881	0.342	0.88 [0.65–1.05]	0.297	0.351	0.204	0.31	0.617
*CLOCK* MM *n* = 46	0.983	0.326	0.94 [0.73–1.22]	0.596				

*Note:* Values are presented as mean, SD, and median [interquartile range] of relative gene expression calculated using the 2^−ΔΔCt^ method. *p*‐values, effect sizes (Cohen's *d*), and AUCs refer to comparisons between MM and control groups and are therefore reported once per gene. Group comparisons were performed using Welch's *t*‐test and Mann–Whitney *U* test, as appropriate.

Abbreviation: AUC, area under the receiver operating characteristic curve.

### 3.2 Diagnostic Performance of *BMAL1* and *CLOCK*

**FIGURE 2:**
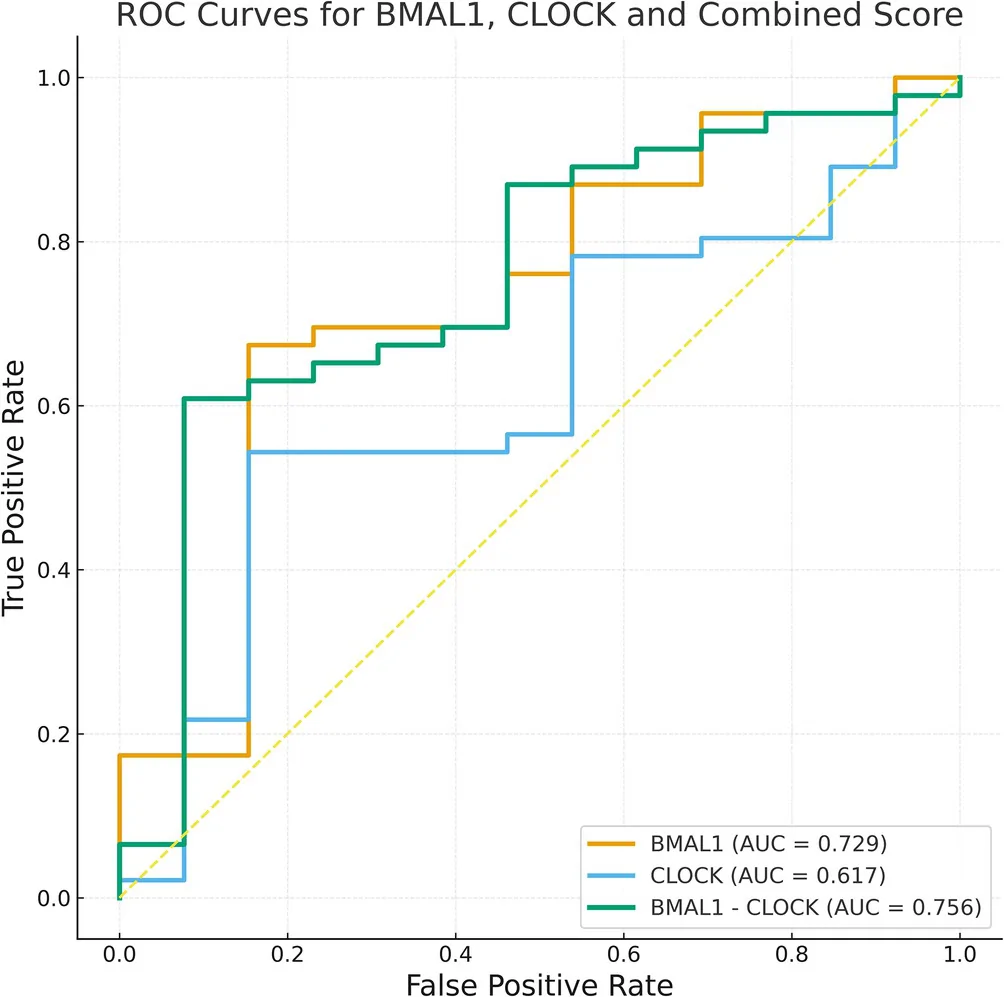
Receiver operating characteristic (ROC) curves showing the diagnostic performance of *BMAL1* and *CLOCK* expression levels in distinguishing multiple myeloma (MM) patients from healthy controls. The diagonal dashed line represents the line of no discrimination (AUC = 0.5).

Receiver‐operating‐characteristic (ROC) analyses confirmed the moderate diagnostic power of *BMAL1* (AUC = 0.729) and the limited discriminative value of *CLOCK* (AUC = 0.617). When both genes were entered into a logistic model, diagnostic accuracy improved slightly (combined AUC = 0.756), highlighting the potential complementary value of their joint evaluation (Figure 2).

### 3.3 Multivariate Logistic Regression Analysis

A multivariate logistic regression model was constructed to evaluate the combined contribution of *BMAL1* and *CLOCK* expression to the odds of Multiple Myeloma status (Table 3; Figures S1 and S2). In this model, *BMAL1* remained a strong independent predictor (*β* = 8.11, *p* = 0.0046; OR = 3343.4, 95% CI 12.2–9.1 × 10^5^), whereas *CLOCK* expression showed an inverse association with MM probability after adjustment for *BMAL1* expression (*β* = −5.58, *p* = 0.019; OR = 0.0037, 95% CI 3.5 × 10^−5^–0.398). The corresponding forest plot (Figure S1) illustrates these effect sizes. Given the strong *BMAL1–CLOCK* coupling, the direction of the adjusted CLOCK coefficient should be interpreted cautiously as an adjusted association rather than as evidence of uniform *CLOCK* downregulation in MM. Thus, the inverse adjusted CLOCK coefficient is most plausibly interpreted as a model‐dependent residual effect (BMAL1‐independent component) rather than a direct expression decrease. Model calibration analysis (Figure S2) demonstrated a good agreement between predicted and observed probabilities, supporting the model's reliability and discriminative capacity. Overall, the multivariable model supports a reproducible BMAL1‐associated MM signal; however, model‐derived diagnostic performance should be interpreted cautiously and requires external validation.

**TABLE 3:** Logistic regression analysis of *BMAL1* and *CLOCK* gene expression for multiple myeloma diagnosis.

Variable	Coef (*β*)	SE	Wald z	*p*	Odds ratio (OR)	95% CI lower	95% CI upper
*BMAL1*	8.11	2.85	2.84	**0.0046**	3343.4	12.2	913 917
*CLOCK*	−5.58	2.39	−2.34	**0.019**	0.0037	0.000035	0.398
Constant	−1.40	1.21	−1.16	0.252	0.25	0.02	2.70

*Note:* Logistic regression model included *BMAL1* and *CLOCK* expression as independent variables to predict multiple myeloma (MM) status. Odds ratios (OR) are exponentiated coefficients. Significant associations (*p* < 0.05) are shown in bold.

Collectively, these findings support *BMAL1* upregulation and an altered *BMAL1/CLOCK* balance in MM at a fixed morning time point; however, because expression was measured in bulk bone marrow, the cellular origin of the signal (CD138+ plasma cells versus microenvironmental compartments) requires cell‐type–resolved validation.

### 3.4 Inter‐Gene Correlation

Spearman correlation analysis revealed a strong positive relationship between *BMAL1* and *CLOCK* expression in both cohorts (*r* = 0.846, *p* = 0.0003 in controls; *r* = 0.796, *p* < 0.0001 in MM), indicating that transcriptional coupling between these clock genes is preserved, although modestly attenuated in MM. As shown in Figure S3, a bivariate scatter distribution of *BMAL1* and *CLOCK* expression highlights this relationship, where 95% confidence ellipses delineate distinct but overlapping patterns for MM and control groups.

### 3.5 Association Between *BMAL1* and *CLOCK* Expression and Clinical Characteristics in MM

The associations between *BMAL1* and *CLOCK* expression levels and clinical as well as laboratory characteristics of multiple myeloma (MM) patients are summarized in Table S2. No significant differences in *BMAL1* or *CLOCK* expression were observed according to age, sex, ISS stage, Durie–Salmon stage, serum albumin, LDH, CRP, calcium, creatinine, hemoglobin levels, or immunoglobulin heavy or light chain types (all *p* > 0.05).

When stratified by β_₂_‐microglobulin levels, *BMAL1* expression showed a non‐significant upward trend with increasing β_₂_‐microglobulin categories (median [IQR]: < 3.5 mg/L, 1.02 [0.80–1.15]; 3.5–5.5 mg/L, 1.17 [0.96–1.36]; > 5.5 mg/L, 1.19 [1.15–1.33]; Kruskal–Wallis *p* = 0.069), suggesting a potential association with disease burden (Figure S4). In contrast, *CLOCK* expression remained stable across β_₂_‐microglobulin categories (Kruskal–Wallis *p* = 0.340), indicating that *CLOCK* expression is independent of tumor burden as reflected by β_₂_‐microglobulin levels.

### 3.6 Partial Correlation Between *BMAL1* and *CLOCK* Expression

To determine whether the association between *BMAL1* and *CLOCK* expression was independent of major clinical covariates, a partial correlation analysis was performed while controlling for age (≤ 65 vs. > 65 years), ISS stage, and β2‐microglobulin categories. Even after adjustment for these prognostic variables, *BMAL1* and *CLOCK* expression levels remained strongly correlated (partial *r* = 0.847, *p* = 2.2 × 10^−13^), indicating that their transcriptional coupling is not attributable to disease stage, tumor burden, or demographic factors. The partial residuals with the 95% confidence ellipse illustrating this relationship are shown in Figure S5.

### 3.7 Machine‐Learning–Based Classification Using *BMAL1* and *CLOCK* Expression

To explore the diagnostic utility of the circadian signature, we trained three supervised classifiers based solely on *BMAL1* and *CLOCK* expression (2^−ΔΔCt^) to discriminate MM cases (*n* = 46) from healthy controls (*n* = 13). Logistic regression, random forest, and support‐vector machine (RBF kernel) models were evaluated using 5‐fold stratified cross‐validation. As summarized in Table S3, the random forest achieved the best overall performance, with an AUC of 0.832, an accuracy of 0.814, a sensitivity of 0.935, and a specificity of 0.385 (Brier score = 0.142). Logistic regression showed moderate discrimination (AUC = 0.747, accuracy = 0.797) with very high sensitivity (0.978) but low specificity (0.154). The SVM model yielded an AUC of 0.691 and, while perfectly sensitive (1.000), failed to correctly classify any control samples (specificity = 0.000), indicating a strong bias toward predicting the MM class. In a class‐weighted sensitivity analysis (Table S4), random forest retained the highest performance (AUC = 0.842; AUPRC = 0.955), whereas class‐weighted logistic regression markedly increased specificity (0.769) but reduced sensitivity (0.652), indicating an expected trade‐off under imbalance correction.

ROC curves for the three classifiers are depicted in Figure S6. Notably, despite moderate AUC values, specificity remained low across models, emphasizing that the two‐gene classifier is exploratory and not suitable for stand‐alone clinical decision‐making in its current form. Consistent with the numerical metrics, the random forest curve lies above those of logistic regression and SVM over most of the false‐positive range, indicating that the two‐gene circadian signature provides only moderate but non‐trivial diagnostic information, with tree‐based modeling extracting slightly more discriminative power than linear or kernel‐based approaches.

### 3.8 Precision–Recall Performance of Machine‐Learning Classifiers Based on *BMAL1* and *CLOCK* Expression

Precision–Recall (PR) analysis was additionally performed to better characterize classifier performance under class imbalance, given the predominance of MM cases in our dataset (46 MM vs. 13 controls). Consistent with the ROC analysis, PR curves demonstrated that models trained exclusively on *BMAL1* and *CLOCK* expression retained moderate but meaningful diagnostic capability.

Among the three evaluated classifiers, the random forest model achieved the highest area under the PR curve (AUPRC = 0.952), reflecting its superior ability to correctly identify MM samples while maintaining a relatively low false‐positive burden. Logistic regression also showed robust performance (AUPRC = 0.896), driven largely by its very high sensitivity but reduced specificity. The SVM (RBF) classifier displayed a lower but still acceptable PR performance (AUPRC = 0.882), consistent with its known tendency to favor the majority class in imbalanced datasets (Figure S7).

Overall, the PR analysis reinforces that a simple two‐gene circadian signature‐comprising *BMAL1* and *CLOCK*‐ is capable of providing non‐trivial discriminative power for distinguishing MM from healthy controls, with tree‐based modeling extracting the highest predictive value. These findings complement the ROC‐based assessment and highlight the biological relevance of circadian deregulation as a potential diagnostic molecular pattern in MM.

## 4 Discussion

Several epidemiological studies suggest that circadian rhythm disruption—particularly in the context of night‐shift work or altered sleep–wake patterns—may influence cancer susceptibility, including endometrial and prostate cancers, as well as certain hematologic malignancies such as chronic lymphocytic leukemia and non‐Hodgkin lymphoma [21, 22]. Beyond exogenous circadian disruption, myeloma biology itself appears to be tightly linked to time‐dependent processes. Recent longitudinal genomic profiling has shown that MM follows a dynamic evolutionary trajectory, with temporal shifts in clonal architecture shaping clinical course and treatment response [8]. In parallel, an immune age–based metric was reported to predict outcomes in older patients receiving daratumumab‐based therapy, underscoring that biological “aging” and immune timing can meaningfully influence treatment response in MM [9]. Together, these observations strengthen the rationale for investigating endogenous clock regulation in MM, particularly the core transcriptional drivers BMAL1 and CLOCK. In this context, the expression patterns of the core positive‐limb genes BMAL1 and CLOCK in MM bone marrow—and their potential diagnostic relevance—have not been systematically evaluated. At the mechanistic level, the circadian clock is increasingly recognized as a regulatory layer that intersects with oncogenic signaling, metabolism, DNA repair, cell‐cycle control, apoptosis, and immune regulation, thereby shaping multiple hallmarks of cancer [11, 12]. In B‐cell tumors, circadian deregulation has also been highlighted as a biologically meaningful axis with potential implications for tumor evolution and therapeutic vulnerability [18]. Despite this broader framework, evidence linking the expression patterns of the core positive‐limb genes *BMAL1* and *CLOCK* to multiple myeloma (MM) has remained limited. Therefore, in the present cohort, we quantified *BMAL1* and *CLOCK* mRNA expression in newly diagnosed MM patients and evaluated their clinical associations and diagnostic potential.

A principal finding of our study is that BMAL1 expression was significantly elevated in MM compared with healthy controls, whereas CLOCK showed only a modest, statistically non‐significant increase. These results are consistent with the concept that circadian circuitry may be dysregulated in cancer, but not necessarily through uniform shifts across all clock genes [11, 12]. Given the limited number of controls (*n* = 13), the study may have reduced sensitivity to detect small‐to‐moderate differences in *CLOCK* expression; therefore, the non‐significant *CLOCK* result should not be interpreted as definitive evidence of no difference. In contrast, *BMAL1* showed a large effect size (Cohen's *d* = 0.83) and remained robust across complementary analyses. In other tumor contexts, BMAL1 has been linked to cancer‐relevant phenotypes, including enhanced proliferation, invasion, and migration. For instance, BMAL1 has been reported to promote colorectal cancer cell migration and invasion through ERK/JNK‐dependent c‐Myc induction [23], and to facilitate breast cancer invasion/metastasis through upregulation of MMP9 [24]. Although these mechanistic data derive from solid tumor systems, they provide a plausible biological basis for why increased BMAL1 expression in MM bone marrow could reflect (or contribute to) an oncogenic transcriptional state.

An important nuance in our dataset is the strong transcriptional coupling between BMAL1 and CLOCK, which remained evident both in MM and in controls. This aligns with the established molecular architecture of the circadian oscillator, in which BMAL1 and CLOCK function as a heterodimeric transcription factor complex that drives rhythmic gene expression, while PER/CRY proteins provide negative feedback to close the loop [11, 12]. Notably, our correlation analyses indicated that BMAL1–CLOCK co‐regulation persists as a robust feature even after accounting for key clinical covariates in MM. This observation supports the interpretation that the MM signal is unlikely to reflect complete silencing of CLOCK; rather, it may involve a shift in the joint BMAL1/CLOCK balance, potentially altering the functional output of the heterodimer and downstream clock‐controlled programs relevant to proliferation and survival [11, 12, 25].

This interpretation becomes particularly relevant when considering our multivariate findings. While CLOCK was not significantly different in univariate comparisons, multivariate logistic regression revealed a strong positive association between BMAL1 and MM status and an inverse adjusted CLOCK coefficient after accounting for BMAL1. In practical terms, this pattern is consistent with a suppression/collinearity effect that can occur when two highly correlated predictors are modeled together: the CLOCK coefficient may reflect the BMAL1‐independent (residual) component of CLOCK rather than a direct group‐wise decrease in CLOCK expression. Accordingly, these results are best interpreted as indicating an altered balance within the BMAL1/CLOCK regulatory unit that may carry exploratory diagnostic information, rather than implying global CLOCK downregulation in MM. The broader literature supports the plausibility that clock gene dysregulation affects cell‐cycle and survival programs in hematologic malignancies; for example, core clock machinery has been shown to regulate leukemia stem cell biology in AML [25], and altered clock gene expression has been reported in hematologic contexts such as chronic lymphocytic leukemia and other B‐cell neoplasms [17].

From a diagnostic standpoint, our ROC analyses demonstrated moderate discrimination for *BMAL1* and limited discrimination for *CLOCK*, with a modest improvement when both genes were considered jointly. To further interrogate whether a minimal circadian signature carries predictive value, we applied supervised machine‐learning classifiers using only *BMAL1* and *CLOCK* expression. Across models, the performance profile consistently indicated non‐trivial but moderate diagnostic capability, with random forest generally extracting slightly higher discriminative power than linear or kernel‐based approaches. Importantly, because our dataset is imbalanced (MM cases outnumber controls), we complemented ROC evaluation with precision–recall analysis, which is more sensitive to class imbalance and highlights the trade‐off between identifying MM cases and avoiding false positives. In this setting, the random forest again achieved the strongest overall PR performance, supporting the idea that a two‐gene circadian signal contains exploratory diagnostic information, while also underscoring that specificity and generalizability will likely require validation in larger, more balanced cohorts. Consistently, in class‐weighted cross‐validation (Table S4), overall discrimination remained moderate while error balance shifted—most notably, logistic regression improved specificity (0.769) at the expense of sensitivity (0.652)—supporting that imbalance‐aware training changes the sensitivity–specificity trade‐off rather than providing a definitive clinical classifier.

We also examined whether *BMAL1* and *CLOCK* expression levels were associated with clinicopathological features in MM. Overall, expression levels did not differ significantly across most clinical subgroups, suggesting that BMAL1/CLOCK expression pattern may reflect a disease‐associated molecular pattern rather than a simple surrogate of standard clinical variables. Nevertheless, we observed a trend toward higher *BMAL1* expression in patients with elevated β2‐microglobulin, which may be clinically relevant because β2‐microglobulin is widely used as an indicator of tumor burden and prognosis in MM. Although our study was not powered to establish definitive prognostic relationships, this trend raises the possibility that *BMAL1* upregulation could track with disease burden in a subset of patients and should be explored in larger series and, ideally, longitudinal sampling.

Our results can also be interpreted alongside emerging MM‐specific evidence implicating circadian pathways. A recent genetic study suggested that polymorphisms in the clock gene PER3 may influence MM susceptibility [19], supporting the concept that inherited variation in circadian regulation can intersect with myeloma biology. Additionally, pharmacologic modulation of circadian regulators has demonstrated anti‐myeloma activity: activation of REV‐ERB with the agonist SR9009 was reported to exert synergistic antitumor effects in MM models by suppressing GRP78‐dependent autophagy and lipogenesis [20]. Together with the broader oncologic literature linking circadian disruption to tumor phenotypes and treatment vulnerability [11, 12, 18], these MM‐focused studies support the relevance of circadian circuitry as a biological dimension worth deeper investigation in myeloma.

Several limitations should be acknowledged. First, the relatively small number of controls may constrain specificity estimates and can influence classifier behavior under class imbalance; external validation in independent cohorts is therefore essential. Controls were derived from individuals undergoing cardiac surgery, a setting that may involve systemic stress and inflammatory activation capable of influencing circadian gene expression. Although screening/exclusion criteria were applied to reduce baseline confounding, we cannot exclude perioperative physiologic effects as a source of bias in baseline expression estimates. Second, our analyses are based on mRNA measurements from bone marrow samples, which may reflect both malignant and non‐malignant cellular contributions; future work incorporating cell‐type–resolved approaches (e.g., CD138+ plasma cells) would strengthen biological interpretation. Finally, although sampling was standardized to a morning time window, circadian transcripts can exhibit time‐of‐day variation; replication with repeated sampling or strict chronobiological controls may further refine the observed signal Because only a single morning time point was analyzed, we cannot distinguish amplitude changes from phase shifts and do not claim loss of rhythmicity. Bulk marrow profiling does not localize the BMAL1 signal to CD138+ malignant plasma cells versus the microenvironment; therefore, cell‐type–resolved validation is needed to define the cellular source of the observed differences. Normalization relied on a single reference gene (*ACTB*); while widely used, future studies should incorporate multiple reference genes and geometric‐mean normalization to improve robustness in bone marrow contexts.

In conclusion, our study indicates that *BMAL1* is significantly upregulated in MM, whereas *CLOCK* alone shows limited discriminatory value. When considered jointly, the *BMAL1/CLOCK* axis provides a moderate, exploratory diagnostic signal across both classical statistical analyses and machine‐learning models, while underscoring the need for validation in larger, independent cohorts. Together with emerging MM literature on clock‐gene susceptibility signals [19] and pharmacologic targeting of circadian regulators [20], these findings support the circadian clock machinery as a relevant molecular framework for further mechanistic and translational studies in multiple myeloma [11, 12, 18].

## Author Contributions

Conceptualization: M.E.A., H.A., A.T. Methodology: M.E.A., H.A., K.Ç., T.K. Validation: M.E.A., Ö.İ.A. Formal analysis: M.E.A., H.A., A.T. Investigation: M.E.A., H.A. Writing – original draft: M.E.A. Writing – review and editing: A.T., Ö.İ.A. Visualization: M.E.A. Supervision: A.T., M.E.E. All authors have reviewed the manuscript and approved the final version for submission and publication.

## Funding

This research was financially supported by the Scientific Research Projects Coordination Unit of Mersin University (2020‐1‐TP2‐4041).

## Ethics Statement

The study protocol was designed and conducted in accordance with the ethical principles outlined in the Declaration of Helsinki (as revised in 2013). Ethical approval was obtained from the Mersin University Clinical Research Ethics Committee prior to the initiation of the study (approval code: 123, date of approval: 19 February 2020).

## Consent

Written informed consent was obtained from all participants before their inclusion in the study.

## Conflicts of Interest

The authors declare no conflicts of interest.

## Supporting information


**Figure S1:** Forest plot illustrating odds ratios (OR) and 95% confidence intervals for *BMAL1* and *CLOCK* expression in the multivariate logistic regression model. *BMAL1* shows a strong positive association with Multiple Myeloma (MM) risk, whereas *CLOCK* displays an inverse relationship. The vertical dashed line represents the null value (OR = 1).
**Figure S2:** Calibration plot of the multivariate logistic regression model assessing *BMAL1* and *CLOCK* expression in relation to MM risk. The solid line depicts the agreement between predicted and observed MM probabilities, while the dashed diagonal line represents the line of perfect calibration. The close alignment of the model curve with the reference line indicates good calibration and predictive reliability.
**Figure S3:** Bivariate scatter plot showing the relationship between *BMAL1* and *CLOCK* expression levels (2^−ΔΔCt^) in multiple myeloma (MM) and control samples. Each dot represents one subject; shaded ellipses indicate 95% confidence intervals for each group. A strong positive correlation was observed in both cohorts (*ρ* = 0.80, *p* < 0.001 in MM; *ρ* = 0.85, *p* < 0.001 in controls). Lines represent fitted trends for visualization purposes.
**Figure S4:** Association of *BMAL1* and *CLOCK* expression with β_₂_‐microglobulin levels in multiple myeloma patients. Boxplots show relative expression levels of *BMAL1* (left) and *CLOCK* (right) (2^−ΔΔCt^) stratified by β_₂_‐microglobulin categories (< 3.5, 3.5–5.5, and > 5.5 mg/L). Group comparisons were performed using the Kruskal‐Wallis test. *BMAL1* expression demonstrated a non‐significant increasing trend with higher β_₂_‐microglobulin levels (*p* = 0.069), whereas *CLOCK* expression did not differ across categories (*p* = 0.340).
**Figure S5:** Partial correlation plot of *BMAL1* and *CLOCK* expression in multiple myeloma. Scatter plot of *BMAL1* and *CLOCK* residuals after removing the linear effects of age, ISS stage, and β2‐microglobulin. Each point represents an individual MM patient. The 95% confidence ellipse indicates a strong positive association (partial *r* = 0.847, *p* = 2.2 × 10^−13^), demonstrating that BMAL1–CLOCK coupling persists independently of clinical covariates.
**Figure S6:** ROC curves of machine‐learning classifiers based on *BMAL1* and *CLOCK* expression. Receiver‐operating‐characteristic (ROC) curves for logistic regression, random forest, and SVM (RBF kernel) models trained on *BMAL1* and *CLOCK* expression (2^−ΔΔCt^) to discriminate Multiple Myeloma (MM) patients (*n* = 46) from healthy controls (*n* = 13). Curves were obtained from out‐of‐fold predicted probabilities using 5‐fold stratified cross‐validation. The dashed diagonal line indicates the line of no discrimination (AUC = 0.5). AUC values for each classifier are shown in the legend.
**Figure S7:** Precision–Recall (PR) curves of machine‐learning classifiers trained on *BMAL1* and *CLOCK* expression (2^−ΔΔCt^). Curves for logistic regression, random forest, and SVM (RBF kernel) models were generated using stratified 5‐fold cross‐validation. The random forest showed the highest precision–recall performance (AUPRC = 0.952), followed by logistic regression (AUPRC = 0.896) and SVM (AUPRC = 0.882). The superior PR performance of the random forest classifier indicates more effective handling of dataset imbalance and improved identification of MM cases.

**Table S1:** Primer and probe sequences used in this study.
**Table S2:** Association of *BMAL1* and *CLOCK* expression with clinical and laboratory parameters in MM patients.
**Table S3:** Performance of machine‐learning classifiers based on *BMAL1* and *CLOCK* expression.
**Table S4:** Sensitivity analysis of class‐weighted machine‐learning classifiers based on BMAL1 and CLOCK expression.

## Data Availability

The data that support the findings of this study are available on request from the corresponding author. The data are not publicly available due to privacy or ethical restrictions.
