# Supplementary material for: Circadian Deregulation in Multiple Myeloma: BMAL1/CLOCK Expression Patterns and Diagnostic Performance
Source: Int J Lab Hematol. 2026 May 21;48(5):1123–32. doi: 10.1111/ijlh.70154 (PMC13555196; doi:10.1111/ijlh.70154)
Supplement: Supplementary file 1 [file IJLH-48-1123-s001.docx]

Table S1. Primer and probe sequences used in this study.

| **Gene** | **Primers and probe sequences** |
| --- | --- |
| ***BMAL1***  *(basic helix-loop-helix ARNT like 1*, Gene ID:406) *** | Forward (F): 5’-CGCAATTGGACGACTGCAT-3’  Reverse (R): 5’-CATATTCCATAGATTTCACCCTGATTT-3’  Probe (Prob): 5’-FAM-CTCATGTAGTTCCACAACCAGTGAACGGG-BHQ1-3’ |
| ***CLOCK***  *(clock circadian regulator,* Gene ID:9575) *** | Forward (F): 5’-GCAGCCGGAAGCATGGT-3’  Reverse (R): 5’-AATCTTATCTGCCTGTCCTGAGTGA-3’  Probe (Prob): 5’-FAM-CCATCTAGTATGCCACAAAACAGCACCCA-BHQ1-3’ |
| ***β-actin (ACTB)***  *(actin beta,* Gene ID: 60) * | Forward (F): 5’-GGCACCCAGCACAATGAAG-3’  Reverse (R): 5’-GCCGATCCACACGGAGTACT-3’  Probe (Prob): 5’-Yakima Yellow-TCAAGATCATTGCTCCTCCTGAGCGC-BHQ1-3 |

*NCBI Gene database (National Center for Biotechnology Information, Bethesda, MD, USA; https://www.ncbi.nlm.nih.gov/gene; accessed December 17, 2025)

Table S2. Association of *BMAL1* and *CLOCK* expression with clinical and laboratory parameters in MM patients.

| **Variable** | ***BMAL1* Median [IQR] by category** | **p**  ***BMAL1*** | **Test** | ***CLOCK* Median [IQR] by category** | **p**  **CLOCK** | **Test** |
| --- | --- | --- | --- | --- | --- | --- |
| **Age** | <=65: 1.12 [0.85–1.25] (*n*=25)  >65: 1.09 [0.95–1.37] (*n*=21) | 0.965 | MWU | <=65: 1.03 [0.72–1.18] (*n*=25)  >65: 0.95 [0.76–1.32] (*n*=21) | 0.791 | MWU |
| **Sex** | Female: 1.06 [0.85–1.20] (*n*=19)  Male: 1.16 [1.02–1.34] (*n*=27) | 0.274 | MWU | Female: 0.88 [0.72–1.19] (n=19)  Male: 1.07 [0.80–1.26] (*n*=27) | 0.435 | MWU |
| **ISS_R** | Stage I: 1.10 [0.88–1.22] (*n*=6)  Stage II: 1.09 [0.87–1.26] (*n*=34)  Stage III: 1.26 [1.17–1.52] (*n*=6) | 0.167 | KW | Stage I: 1.03 [0.83–1.15] (*n*=6)  Stage II: 0.99 [0.70–1.19] (*n*=34)  Stage III: 1.24 [1.00–1.38] (*n*=6) | 0.178 | KW |
| **Durie**  **Salmon**  **Stage** | 1: 1.12 [0.85–1.37] (*n*=16) 2: 1.17 [1.04–1.28] (*n*=20) 3: 1.02 [0.91–1.11] (*n*=10) | 0.520 | KW | 1: 1.10 [0.76–1.19] (*n*=16)  2: 1.05 [0.85–1.29] (*n*=20)  3: 0.77 [0.57–1.15] (*n*=10) | 0.312 | KW |
| **ALB** | Low: 1.12 [0.97–1.43] (*n*=23)  Normal: 1.10 [0.88–1.26] *(n*=23) | 0.429 | MWU | Low: 1.03 [0.80–1.20] (*n*=23)  Normal: 1.06 [0.71–1.21] (*n*=23) | 0.613 | MWU |
| **LDH** | Low: 1.19 [1.10–1.37] (*n*=9)  Normal: 1.07 [0.91–1.25] (*n*=20)  High: 1.12 [0.85–1.25] (*n*=17) | 0.370 | KW | Low: 1.14 [0.95–1.17] (*n*=9)  Normal: 0.95 [0.66–1.21] (*n*=20)  High: 1.03 [0.72–1.30] (*n*=17) | 0.698 | KW |
| **CRP** | >5: 1.16 [1.00–1.37] (*n*=24)  ≤5: 1.10 [0.91–1.25] (*n*=22) | 0.339 | MWU | >5: 1.05 [0.76–1.20] (*n*=24)  ≤5: 0.99 [0.68–1.21] (*n*=22) | 0.605 | MWU |
| **β2-MG** | <3.5–5.5: 1.17 [0.96–1.36] (*n*=19)  <3.5: 1.02 [0.80–1.15] (*n*=18)  >5.5: 1.19 [1.15–1.33] (*n*=9) | 0.069 | KW | 3.5–5.5: 1.02 [0.77–1.18] (*n*=19)  3.5: 0.95 [0.63–1.20] (*n*=18)  >5.5: 1.12 [0.94–1.30] (*n*=9) | 0.340 | KW |
| **Ca** | High: 1.16 [1.11–1.26] (*n*=3)  Low: 1.25 [1.09–1.43] (*n*=9)  Normal: 1.09 [0.87–1.25] (*n*=34) | 0.400 | KW | High: 0.94 [0.90–1.05] (*n*=3)  Low: 1.07 [1.02–1.28] (*n*=9)  Normal: 0.99 [0.70–1.20] (*n*=34) | 0.617 | KW |
| **CRE** | >0.9: 1.17 [1.05–1.36] (n=26)  ≤0.9: 1.09 [0.79–1.25] (*n*=20) | 0.108 | MWU | >0.9: 1.07 [0.85–1.26] (*n*=26)  ≤0.9: 0.95 [0.66–1.17] (*n*=20) | 0.166 | MWU |
| **Hb** | Low: 1.12 [1.02–1.34] (*n*=22)  Normal: 1.10 [0.80–1.24] (*n*=24) | 0.312 | MWU | Low: 1.03 [0.84–1.20] (*n*=23)  Normal: 0.94 [0.72–1.21] (*n*=23) | 0.742 | MWU |
| **Ig Type** | IgA: 1.17 [0.85–1.25] (*n*=9)  IgG: 1.09 [0.93–1.33] (*n*=37) | 0.868 | MWU | IgA: 0.84 [0.72–1.17] (*n*=9) IgG: 1.06 [0.76–1.21] (*n*=37) | 0.391 | MWU |
| **Ig Light Chain** | Kappa: 1.13 [0.85–1.28] (n=32)  Lambda: 1.09 [1.00–1.29] (*n*=14) | 0.990 | MWU | Kappa: 1.02 [0.71–1.19] (*n*=32)  Lambda: 1.12 [0.84–1.35] (*n*=14) | 0.257 | MWU |

MWU: Mann-Whitney U; KW:Kruskal-Wallis. Values are shown as median [IQR] of 2^-ΔΔCt^ per category with sample sizes in parentheses. Group comparisons used Mann–Whitney U (2 groups) or Kruskal-Wallis (≥3 groups). Significance set at p<0.05 (two-tailed).

Table S3. Performance of machine-learning classifiers based on *BMAL1* and *CLOCK* expression.

| **Model** | **AUC (ROC)** | **AUPRC** | **Accuracy** | **Sensitivity** | **Specificity** | **Brier score** |
| --- | --- | --- | --- | --- | --- | --- |
| Logistic regression | 0.747 | 0.896 | 0.797 | 0.978 | 0.154 | 0.168 |
| Random forest | 0.832 | 0.952 | 0.814 | 0.935 | 0.385 | 0.142 |
| SVM (RBF kernel) | 0.691 | 0.882 | 0.780 | 1.000 | 0.000 | 0.214 |

Performance metrics of supervised machine-learning classifiers trained on *BMAL1* and *CLOCK* expression values (2^−ΔΔCt^) to discriminate multiple myeloma (MM) patients (*n* = 46) from healthy controls (*n* = 13). Models were evaluated using stratified 5-fold cross-validation. AUC, area under the ROC curve; AUPRC, area under the precision–recall curve.

Table S4. Sensitivity analysis of class-weighted machine-learning classifiers based on BMAL1 and CLOCK expression.

| **Model** | **Class-weight** | **AUC** | **AUPRC** | **Acc.** | **Sens.** | **Spec.** | **Brier** |
| --- | --- | --- | --- | --- | --- | --- | --- |
| Logistic regression | Balanced | 0.756 | 0.887 | 0.678 | 0.652 | 0.769 | 0.203 |
| Random forest | Balanced-subsample | 0.842 | 0.955 | 0.831 | 0.957 | 0.385 | 0.138 |
| SVM (RBF kernel) | Balanced | 0.645 | 0.847 | 0.780 | 1.000 | 0.000 | 0.168 |

Performance metrics of supervised machine-learning classifiers trained on *BMAL1* and *CLOCK* expression values (2^−ΔΔCt^) to discriminate multiple myeloma (MM) patients (*n* = 46) from healthy controls (*n* = 13). Models were evaluated using stratified 5-fold cross-validation with class-weighted training. AUC, area under the ROC curve; AUPRC, area under the precision–recall curve.
