# Supplementary material for: Circadian Deregulation in Multiple Myeloma: BMAL1/CLOCK Expression Patterns and Diagnostic Performance
Source: Int J Lab Hematol. 2026 May 21;48(5):1123–32. doi: 10.1111/ijlh.70154 (PMC13555196; doi:10.1111/ijlh.70154)
Supplement: Supplementary file 2 [file IJLH-48-1123-s002.docx]

Figure S1. Forest plot illustrating odds ratios (OR) and 95% confidence intervals for *BMAL1* and *CLOCK* expression in the multivariate logistic regression model. *BMAL1* shows a strong positive association with Multiple Myeloma (MM) risk, whereas *CLOCK* displays an inverse relationship. The vertical dashed line represents the null value (OR = 1).

Figure S2. Calibration plot of the multivariate logistic regression model assessing *BMAL1* and *CLOCK* expression in relation to MM risk. The solid line depicts the agreement between predicted and observed MM probabilities, while the dashed diagonal line represents the line of perfect calibration. The close alignment of the model curve with the reference line indicates good calibration and predictive reliability.

Figure S3. Bivariate scatter plot showing the relationship between *BMAL1* and *CLOCK* expression levels (2^–ΔΔCt^) in multiple myeloma (MM) and control samples. Each dot represents one subject; shaded ellipses indicate 95% confidence intervals for each group. A strong positive correlation was observed in both cohorts (ρ = 0.80, p < 0.001 in MM; ρ = 0.85, p < 0.001 in controls). Lines represent fitted trends for visualization purposes.

Figure S4. Association of *BMAL1* and *CLOCK* expression with β₂-microglobulin levels in multiple myeloma patients. Boxplots show relative expression levels of *BMAL1* (left) and *CLOCK* (right) (2^−ΔΔCt^) stratified by β₂-microglobulin categories (<3.5, 3.5–5.5, and >5.5 mg/L). Group comparisons were performed using the Kruskal-Wallis test. *BMAL1* expression demonstrated a non-significant increasing trend with higher β₂-microglobulin levels (p = 0.069), whereas *CLOCK* expression did not differ across categories (p = 0.340).

Figure S5. Partial correlation plot of *BMAL1* and *CLOCK* expression in multiple myeloma. Scatter plot of *BMAL1* and *CLOCK* residuals after removing the linear effects of age, ISS stage, and β2-microglobulin. Each point represents an individual MM patient. The 95% confidence ellipse indicates a strong positive association (partial r = 0.847, p = 2.2 × 10⁻¹³), demonstrating that BMAL1–CLOCK coupling persists independently of clinical covariates.

Figure S6. ROC curves of machine-learning classifiers based on *BMAL1* and *CLOCK* expression. Receiver-operating-characteristic (ROC) curves for logistic regression, random forest, and SVM (RBF kernel) models trained on *BMAL1* and *CLOCK* expression (2^−ΔΔCt^) to discriminate Multiple Myeloma (MM) patients (*n* = 46) from healthy controls (*n* = 13). Curves were obtained from out-of-fold predicted probabilities using 5-fold stratified cross-validation. The dashed diagonal line indicates the line of no discrimination (AUC = 0.5). AUC values for each classifier are shown in the legend.

Figure S7. Precision–Recall (PR) curves of machine-learning classifiers trained on *BMAL1* and *CLOCK* expression (2^−ΔΔCt^). Curves for logistic regression, random forest, and SVM (RBF kernel) models were generated using stratified 5-fold cross-validation. The random forest showed the highest precision–recall performance (AUPRC = 0.952), followed by logistic regression (AUPRC = 0.896) and SVM (AUPRC = 0.882). The superior PR performance of the random forest classifier indicates more effective handling of dataset imbalance and improved identification of MM cases.
